# Medical Opinion and Movement

**Published:** 1907-07-20

**Authors:** 


### Medical Opinion and Movement.

The order of the London County Council for the notification of cerebro-spinal fever for a period of six months has raised the question whether posterior basal meningitis should be included under "cerebro-spinal fever" for purposes of notification. The Public Health Committee of the County Council referred the matter to Sir Douglas Powell, President of the Royal College of Physicians, and he appointed a committee to consider the question. This committee has decided that although there are some differences clinically between the two diseases, in the majority of cases a differentiation is impracticable, and that bacteriologically the organism associated with each disease (Meningococcus intracellulosus) is practically identical. The committee recommends, therefore, that for notification purposes cases of posterior basal meningitis should be included under the term cerebro-spinal fever. They also make two further recommendations, namely: that the order of notification shall continue in force for a period of two years, in order to cover two possible epidemic periods; and that after notification details of the cases should be obtained in hospitals through the courtesy of medical officers in charge, and in private by a special officer available to investigate cases, if desired, in association with the practitioner in charge. The Public Health Committee intends to recommend the County Council to give effect to these suggestions, and in that way the notification of the disease will serve the purpose of furnishing facts which may throw further light upon the etiology of the disease.

One of the most satisfactory facts which is brought out in Dr. Tatham's comparison of vital statistics for the successive decennial periods of the last fifty years, to which we have already referred in a previous issue, is the steady and rapid decline in the mortality from tuberculosis. Comparing the period 1891-1900 with 1851-60, the decline in death-rate from all forms of tuberculosis amounts to nearly 50 per cent. The figures must not be taken too literally as representing the actual state of things, as the diagnosis was undoubtedly not so accurate fifty years ago, and a considerable portion of deaths in the "fifties" and "sixties" were uncertified. The decline has, however, been fairly steady throughout, and has continued since 1900, according to the latest available statistics for 1905, at an even greater pace. This fall in mortality is mainly due to a decrease in deaths from pulmonary disease, and has affected women to a greater extent than men, so that the relation of the two sexes in regard to mortality from tuberculosis has been reversed. Whereas fifty years ago more females died from phthisis than males, now the proportion of deaths is greater in males. This increased mortality for males occurs chiefly in the urban districts, and suggests their segregation in workshops and factories as the probable cause.

It is already sufficiently well known that prolonged exposure to the $x$-rays may result in azoospermia, and that workers with the $x$-rays are especially liable to this condition. Dr. Alfred C. Jordan, however, draws attention to the fact that sterility may result from working daily with the $x$-rays for a year or so, even though the operator has taken "every reasonable care," to avoid exposing himself unduly to their action. By "reasonable care," Dr. Jordan means that he has had his $x$-ray tube enclosed in a protection box, has worn a protective apron, has conducted the $x$-ray treatment with a tube in a protective shield, and has himself kept at a distance of a few yards from the tube. Dr. Albert Schönberg, of Hamburg, adopts the expedient of enclosing himself within a cabin lined with sheet lead and placed in the middle of his radiography room. The switchboard is within the cabin, and he observes the tube and the patient through a small window of lead-glass. Dr. Jordan suggests the means which he thinks should prove efficient to prevent any delete rious effects of the $x$-rays upon the operator. The tube-box should be entirely of wood lined completely on the outside with an efficient protective rubber material, except for an aperture at the top with an adjustable diaphragm. A view of the tube may be obtained through a small lead-glass window covered with a flap of the protective material. The operator should wear a protective apron extending from the shoulders to below the knees. In addition, he should also be provided with upright rectangular wooden screens on castors, lined on both faces with 3lb. sheet lead.

Nearly a year ago a new regulation was issued by the Postmaster-General intimating that in future the efficiency of postal medical officers over sixty years of age would be reviewed annually, only those being retained who were found efficient, in no case but . . . if over seventy years of age. The Association of the British Postal Medical Officers has made strenuous efforts to obtain a recission or modification of this regulation, and is to be congratulated on the success which has attended them. On June 27 a deputation was received by Mr. Buxton from the Association, and it was pointed out that the regulation involved great hardship in being retrospective; that medical officers were given no opportunity of refuting any charges of inefficiency; and that they would be the only servants of the Post Office liable to dismissal for inefficiency without warning, and the only public servants to be retired under an age-limit without compensation or gratuity. In consequence of these representations, Mr. Buxton has consented to considerably modify the original regulations. Present and prospective medical officers over seventy years of age will be favourably considered for retention, when they can be shown to be still efficient. Only hopeless inefficiency will result in dismissal without warning. On the other hand, in cases of slackness, twelve months' notice will be given, and if in that time the medical officer is again reported as efficient, his services will be retained. Furthermore, a scheme of superannuation, to be drawn up by the Association, has been promised the sympathetic consideration of the Postmaster-General.

